# An altered maturation and adhesion phenotype of dendritic cells in diseased individuals compared to asymptomatic carriers of human T-cell leukemia virus type 1

**DOI:** 10.1186/1742-4690-11-S1-O67

**Published:** 2014-01-07

**Authors:** Sharrón L Manuel, Mohit Sehgal, Zafar K Khan, James J Goedert, Michael R Betts, Pooja Jain

**Affiliations:** 1 Drexel Institute for Biotechnology and Virology Research, and the Department of Microbiology and Immunology, Drexel University College of Medicine, Doylestown, PA, USA; 2 National Cancer Institute, Division of Cancer Epidemiology and Genetics, Bethesda, MD, USA; 3 Department of Microbiology and Immunology, University of Pennsylvania School of Medicine, Philadelphia, PA, USA

## 

As critical effectors of antiviral immune response, dendritic cells (DCs) are implicated to play an important role in determining the outcome of HTLV-1 infection. However, a complete understanding of their role in any disease pathogenesis requires extensive assessment of the phenotypic and functional state of DCs. To enable this, we developed a polychromatic antibody cocktail comprising key phenotypic and functional markers of DCs and applied it in a patient cohort from the HTLV-1 endemic region, Jamaica, consisted of seronegative controls, asymptomatic carriers (ACs), ATL, and HAM/TSP patients. This ex vivo analyses included two major subsets of blood DCs, myeloid and plasmacytoid (mDCs and pDCs, respectively). The comparative analyses of results demonstrated a decreased pDC frequency in both ATL and HAM/TSP patients as compared to ACs and seronegative controls. Similarly, CD86 expression on both mDCs and pDCs was significantly higher in HAM/TSP (but not ATL) patients compared to ACs. Interestingly, HLA-DR expression was significantly lower on pDCs of patients as compared to carriers, whereas for mDCs, only the HAM/TSP group had significantly lower expression of HLA-DR. Unlike HAM/TSP individuals, ATL individuals had significantly higher HLA-ABC expression on mDCs compared to ACs. Finally, both mDCs and pDCs of HAM/TSP patients had significantly higher PD-L1 expression compared to ACs. Overall, this study suggests that DCs are differentially regulated between patients (ATL and HAM/TSP) and carriers of HTLV-1 and could provide an important tool to understand HTLV-1 immunopathogenesis during infection and disease.

